# Editorial: Targeting regulatory cells in cancer: old and new approaches in immunotherapy

**DOI:** 10.3389/fimmu.2024.1458280

**Published:** 2024-07-16

**Authors:** Luigi Cari, Rosalinda Sorrentino, Billur Akkaya, Giuseppe Nocentini

**Affiliations:** ^1^ Department of Medicine and Surgery, University of Perugia, Perugia, Italy; ^2^ Department of Pharmacy (DIFARMA), University of Salerno, Salerno, Italy; ^3^ Pelotonia Institute for Immuno-Oncology, The Ohio State University, Columbus, OH, United States; ^4^ Department of Neurology, The Ohio State University Wexner Medical Center, Columbus, OH, United States

**Keywords:** regulatory cells, Treg cells, cell plasticity, tumor microenvironment, immunotherapy, bispecific antibodies, immune checkpoint inhibitors

The tumor microenvironment (TME) plays a crucial role in cancer progression and response to therapeutic interventions. Comprising a complex network of cancer cells, stromal cells, blood vessels, and immune components, the TME significantly impacts the efficacy of treatments. Recent therapeutic strategies have focused on enhancing the activation of the immune system within the TME, such as the activation of CD8^+^ T cells. Despite the potential of these approaches, clinical responses have been observed in only a limited number of patients underscoring the necessity for additional therapeutic strategies (1). One promising avenue is targeting regulatory cells within the TME. This strategy, which can be used alone or in combination with existing immunotherapies, aims to break the immunosuppressive environment that malignancies frequently develop to evade immune surveillance. It has the potential to overcome current limitations and provide more effective treatments for a broader patient population.

Among the regulatory cells infiltrating TME, we find several cells including regulatory T cells (Tregs), myeloid-derived suppressor cells (MDSCs), cancer-associated mesenchymal stem/stromal cells (CA-MSCs). While significant progress has been made in understanding the role of regulatory cells in cancer, the TME is often a significant barrier to successful immunotherapy (2). Targeting regulatory cells can potentially enhance the efficacy of existing immunotherapies and provide new avenues for treatment-resistant cancers. The integration of regulatory cell depletion/inhibition into standard cancer treatment protocols will offer hope for more effective and durable responses in cancer patients.

Attempting to contribute to addressing ongoing and emerging strategies to target regulatory cells present in the TME, this Research Topic, titled “Targeting Regulatory Cells in Cancer: Old and New Approaches in Immunotherapy” not only covers recent advances but also highlights novel approaches. The Research Topic was curated by four guest editors and consists of six articles: three reviews and three original research investigations.

In their article titled “Targeting tumor-infiltrating Tregs for improved antitumor responses” Qin et al. review the phenotypic characteristics and subpopulations of regulatory T cells (Tregs) within the TME, as well as the inhibitory mechanisms these cells employ against antitumor responses. Additionally, the Authors discuss current major strategies for targeting Tregs from the TME, including specific killing, functional inhibition, and reduced recruitment. All these therapeutic strategies are summarized in Table 1 of the manuscript, with a discussion of their respective advantages and limitations.

Still regarding the role of Treg cells in the TME, Song et al., in their article “A new target of radiotherapy combined with immunotherapy: regulatory T cells” discuss how the presence of Treg cells in the TME affects the efficacy of radiation therapy. Radiotherapy alone not only kills tumor cells directly but also regulates the immune response of tumors by reshaping the immune microenvironment, thereby increasing the immunosuppressive function of Tregs. In their manuscript, the Authors review how the combination of radiotherapy and immunotherapy, the last approach increasing Treg depletion and reducing their function, can achieve synergistic effects that benefit patients.

In the article “Targeting regulatory T cells by E7777 enhances CD8^+^ T-cell–mediated anti-tumor activity and extends survival benefit of anti-PD-1 in solid tumor models” Mahdi et al. investigates the therapeutic potential of E7777 (a new formulation of denileukin diftitox) in combination with anti-PD-1 antibodies to improve anti-tumor responses and overall survival in murine models of colon and liver cancer. The authors demonstrates that the treatment was well-tolerated and the presence of Tregs induced by anti-PD-1 is effectively countered by E7777, suggesting a synergistic effect. The study highlights the potential of E7777 in combination with anti-PD-1 therapy to enhance CD8^+^ T-cell-mediated anti-tumor responses and improve survival outcomes in solid tumor models, offering a promising strategy for cancer immunotherapy. Based on the results of this study, a Phase 1/1b study is underway at the University of Pittsburgh Medical Center (UPMC), Hillman Cancer Center (NCT05200559), and a Phase 1 trial has also been initiated at the University of Minnesota, Masonic Cancer Center (NCT04855253).

Going beyond the role of regulatory T cells, the article “Cancer-associated mesenchymal stem/stromal cells: role in progression and potential targets for therapeutic approaches”, by Hazrati et al. shed light on the significant impact of CA-MSCs in tumor progression. The article highlights the importance of understanding how CA-MSCs influence vital aspects of tumor biology, such as cell proliferation, drug resistance, angiogenesis, and tumor cell invasion and metastasis. The review explores the specific mechanisms by which CA-MSCs suppress both the innate and adaptive immune systems, as well as other pathways that contribute to tumor progression, providing insights into the potential therapeutic targeting of CA-MSCs as a strategy to more effectively combat cancer by interfering with the supportive roles these cells play in tumor development and maintenance.

Another novel therapeutic approach has been proposed by Jiang et al. that, in the article “A novel dual mechanism of action bispecific PD-1-IL-2v armed by a “βγ-only” interleukin-2 variant”, explores a novel bispecific PD-1-IL-2v molecule for cancer therapy, showing significant anti-tumor efficacy at high doses. Designed by structural truncation and shuffling, this IL-2 variant activates T and NK cells and reduces Treg activation. The Authors demonstrated that, unlike existing IL-2 variants, the new variant binds selectively to the IL-2R βγ complex, avoiding immune overstimulation while enhancing anti-tumor activity. The molecule also blocks PD-1/L1 interactions, maintaining the stemness of CD8^+^ T and NK cells.

Finally, Rossini et al., in the article “Epacadostat stabilizes the apo-form of IDO1 and signals a protumorigenic pathway in human ovarian cancer cells” demonstrate that the IDO1 inhibitor epacadostat was found to stabilize the apo-form of IDO1, enhancing its association with the phosphatase SHP-2 and activating a signaling pathway that supports the tumorigenic phenotype in SKOV-3 ovarian cancer cells. These results can explain why, despite high IDO1 expression in cancer is associated with poor patient prognosis, IDO1 catalytic inhibitors have not shown the expected anti-tumor efficacy in clinical trials.

We believe that this Research Topic offers an updated perspective and contribute to the development of future therapeutic strategies.

## Author contributions

LC: Writing – original draft, Writing – review & editing. RS: Writing – original draft, Writing – review & editing. BA: Writing – original draft, Writing – review & editing. GN: Writing – original draft, Writing – review & editing.
